# Buspirone in Autism Spectrum Disorder: A Systematic Review

**DOI:** 10.7759/cureus.39304

**Published:** 2023-05-21

**Authors:** Nihit Gupta, Mayank Gupta, Raghu Gandhi

**Affiliations:** 1 Psychiatry, Dayton Children's Hospital, Dayton, USA; 2 Psychiatry and Behavioral Sciences, Southwood Psychiatric Hospital, Pittsburgh, USA; 3 Psychiatry, Abbott Northwestern Hospital, Minneapolis, USA

**Keywords:** neurodevelopmental disorders, autism spectrum disorder and emotion, autism spectrum disorder (asd), buspirone, a systematic review

## Abstract

The aim of this systematic review is to appraise the current evidence on the efficacy and safety of buspirone in core symptoms of autism spectrum disorder (ASD), co-occurring anxiety, and other associated symptoms. Major medical literature databases were searched for randomized controlled trials (RCTs), open-label trials, and any other relevant studies or clinical trials reporting on pediatric (age < 18 years) patients with ASD treated with buspirone for any reason. A total of 310 abstracts were screened, and six clinical trials were selected for inclusion. Out of these six clinical trials, two were RCTs (*n* =166 and 40), two open-label trials (n= 26 and 4), and one cross-over study (*n* = 1). We also included one retrospective chart review (n=31). Meta-analysis was not performed due to a lack of homogeneity in the two RCTs. Although most of the studies reported improved overall symptoms, they had different outcome measures. The quality of evidence available is low, and there is a need for higher-power studies in the future. Most studies suggested that buspirone was well tolerated and safe in pediatric patients with ASD. Based on the data, there is insufficient evidence to make conclusive recommendations on buspirone for improvement in core symptoms of ASD or cooccurring anxiety, irritability, or hyperactivity symptoms in the pediatric population. Given there are limited approved therapies for co-occurring anxiety, buspirone could be used as a safe off-label option due to the lack of behavioral activation and any serious adverse reactions.

## Introduction and background

The Diagnostic and Statistical Manual for Mental Disorder-5 (DSM-5) describes autism spectrum disorder (ASD) as a neurodevelopmental disorder characterized by difficulties with social communication and restricted repetitive behaviors and interests [1]. DSM-5 included previous diagnoses of autism, Asperger's syndrome, childhood disintegration disorder, Rett syndrome, and pervasive developmental disorder not otherwise specified under the single umbrella term of ASD [2]. In the United States, according to the CDC’s morbidity and mortality weekly report, it is estimated that one in 36 individuals under the age of eight years suffers from ASD [3]. Diagnostic challenges and lack of availability of evidence-based interventions have confounded the understanding of ASD.

Multimodal approaches, including psychosocial interventions, are used to treat core symptoms of ASD. Applied behavioral analysis (ABA), speech therapy, occupational therapy, physical therapy, and individual educational programs in special education are all beneficial to the overall outcome of treatment. There is a long waitlist for ABA therapy, and it is not widely available [4]. The effectiveness of occupational therapy is limited, despite its widespread use. There is, therefore, a high expectation that these individuals will be helped by psychopharmacology. There are only two United States FDA-approved medications for irritability associated with ASD, risperidone, and aripiprazole [5].

It is estimated that 63% of people with ASD have co-occurring anxiety and 55% have depression and are also at an increased risk for suicide [6]. Selective serotonin reuptake inhibitors (SSRIs) have limited evidence for use in ASD, with a possible risk of harm in individuals with ASD [7]. Moreover, comorbid illnesses can mask the symptoms of ASD due to diagnostic overshadowing [8]. Buspirone is an agonist of the pre-and post-synaptic serotonergic 1A (5HT1A) receptors that are believed to have an anti-anxiety effect due to the diminished firing of serotonergic neurons, resulting from presynaptic 5HT1A agonism. In 1968, a team at Mead Johnson synthesized buspirone, but it was not patented until 1980. It was originally developed to treat psychoses using the D2 receptor, but it was found to be ineffective. In 1986, Bristol-Myers Squibb gained FDA approval for buspirone as a treatment for generalized anxiety disorder (GAD). However, buspirone is not FDA-approved for treating GAD in youths. The safety and effectiveness of buspirone were evaluated in two placebo-controlled six-week trials involving 559 pediatric patients (between 6 and 17 years of age) with GAD. The doses studied were 7.5 mg to 30 mg twice a day (15-60 mg/day). Neither buspirone nor placebo significantly alleviated symptoms of GAD. There were no safety concerns associated with buspirone in these trials. No long-term safety or efficacy data are available for this population [9]. Considering anxiety is significantly comorbid in individuals with ASD, we reviewed all available evidence on the use, effectiveness, and side effects of buspirone in children with ASD.

Method

Our systematic review and metanalysis were performed according to the PRISMA guidelines. The protocol was registered at PROSPERO: CRD42023397654.

Data sources searched

**Figure 1:**
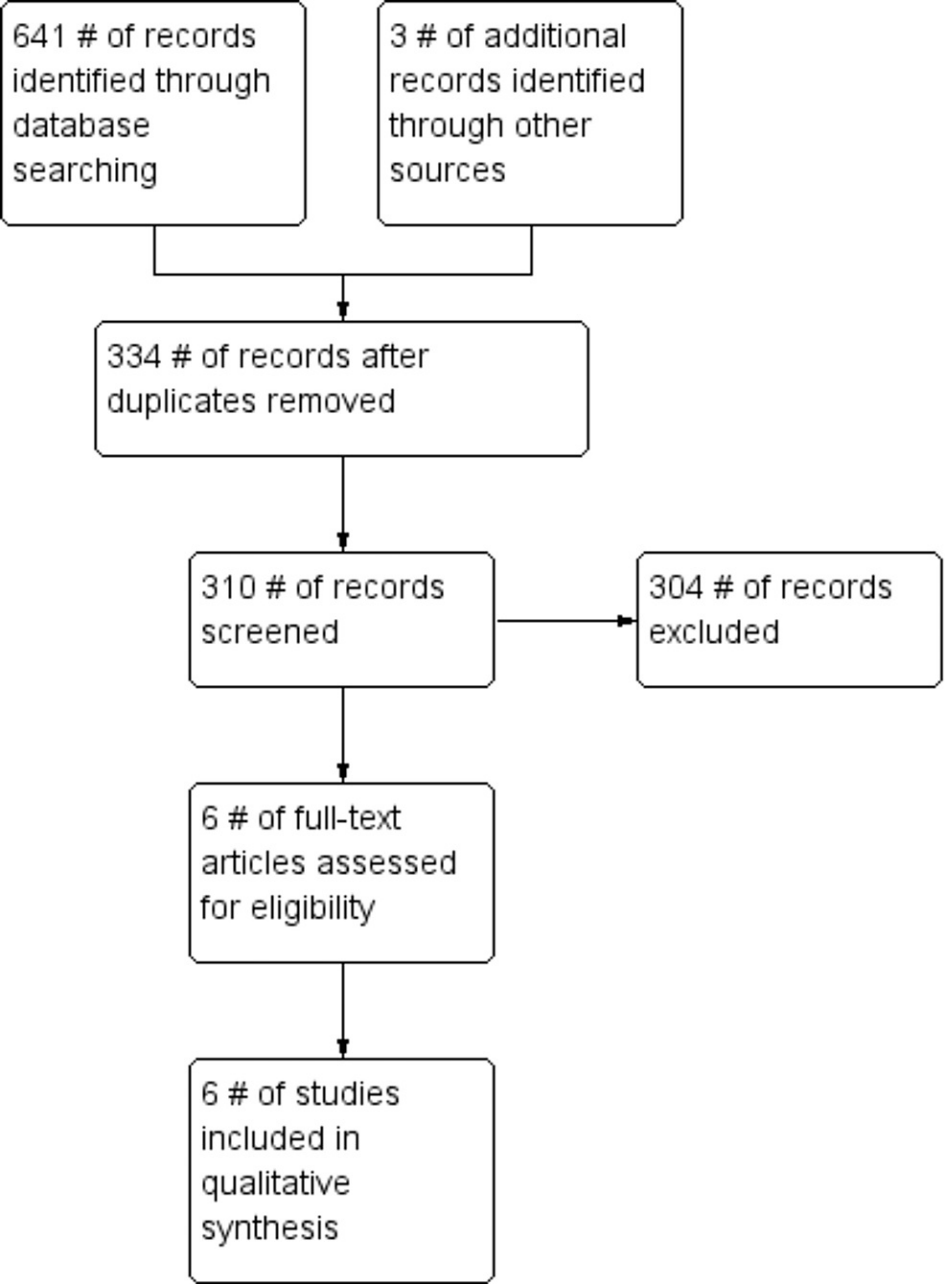
Study flow diagram

A comprehensive search of several databases from each database's inception to 4/1/2023 was conducted. The databases include Pubmed, Scopus, Embase, PsychINFO, Google Scholar, and Web of Science. We also searched for databases of ongoing clinical trials through clinicaltrials.gov. The search was designed by coauthors NG and MG using controlled vocabulary and keywords "autism spectrum disorder," " autistic disorder," "Asperger’s," "PDD-NOS," "neurodevelopmental disorders," and "buspirone.” The search was performed in all languages and was limited to human subjects. The complete search strategy is available in Figure 1. We also performed a manual search of references in included studies to avoid selection bias.

Study selection and eligibility criteria

Two reviewers independently screened the title and abstracts of potentially eligible articles. Subsequently, the full text of eligible articles was reviewed separately by the same two reviewers. The study's inclusion criteria were P: Children under the age of eight years with ASD defined by DSM-5/IV TR with the use of an autism diagnostic observation schedule (ADOS)/ADOS-2. I: Studies evaluating the use of buspirone. C: Treatment as usual including placebo, SSRI, and/or antipsychotics. O: Primary outcome: Improvement in core symptoms of autism. Secondary outcome: Improvement in anxiety and irritability symptoms. S: Clinical trials. Exclusion criteria were any unpublished data.

Data collection

Data was collected from the included studies using a standardized data extraction form by two reviewers (Table 1). We extracted data on the following variables: study characteristics (author, year, country, study design, sample size, demographic characteristics of steady participants, inclusion and exclusion criteria), intervention types, outcome measures, and duration of follow-up.

**Table 1:** Data extraction form RCT: randomized controlled trials, ASD: autism spectrum disorder, HF-ASD: high-functioning autism spectrum disorder, ADOS: autism diagnostic observation schedule, RRBI: restricted and repetitive behaviors and interests, ABC: aberrant behavioral checklist, ADI-R: autism diagnostic interview-revised, PDD: pervasive developmental disorder, CGI: clinical global impression scale, DSM: diagnostic and statistical manual of mental disorders, SMBC: sensory-motor behavioral checklist, SAI: social awareness inventory, CTRS: Conners' teacher rating scale

Author, year, country	Type of study	Diagnosis	Diagnostic tool used	N	Age	Control group	Oral buspirone dose (mg)	Duration	Outcome measures
Chugani et al., 2016, USA [10]	RCT	ASD	ADOS/ABC	166	2-6 yrs	Orasweet liquid	2.5 mg and 5 mg	24 weeks and 48 weeks	ADOS composite scores and RRBI scores
Ghanizadeh et al., 2015, Iran [11]	RCT	ASD	ADI-R/ABC	40	4-17 yrs	Risperidone only	10-20 mg BID along with risperidone	8 weeks	ABC-CRS
Buitelaar et al. 1998., Netherlands [12]	Open label	PDD	DSM-III-R	26	6-17 yrs	n/a	15-45 mg/kg	6-8 weeks with follow-up at 12 months	CGI scale
Realmuto et al. 1989., USA [13]	Open trial	Autistic disorder	DSM-III	4	9-10.5 yrs	Methylphenidate and fenfluramine	5 mg BID	4 weeks	ABC, SMBC, SAI, and CTRS
Mccormick et al. 1997., USA [14]	DB-PC cross over	ASD	DSM-III-R	1	4 yrs 10 months	Lactose hydrose 5 mg	5 mg	3 weeks	Hyperactivity
Ceranoglu et al, 2019., USA [15]	Retrospective chart review	HF-ASD	DSM5	31	8-17 yrs	n/a	24.1 to 41.61 mg/day	125-272 days	CGI-S and CGI-I

Methodological quality and risk of bias assessment

We used the Cochrane Collaboration tool (Cochrane, London, England) for assessing the risk of bias to evaluate the methodological quality of randomized controlled trials (RCTs). We assessed the risk of bias for random sequence generation, allocation concealment, blinding of participants and personnel, blinding of outcome assessment, incomplete outcome data, selective reporting, and other biases (Figures 3-4). For non-randomized/observational studies, the methodological quality was assessed using the methodological index for non-randomized studies.

**Figure 2:**
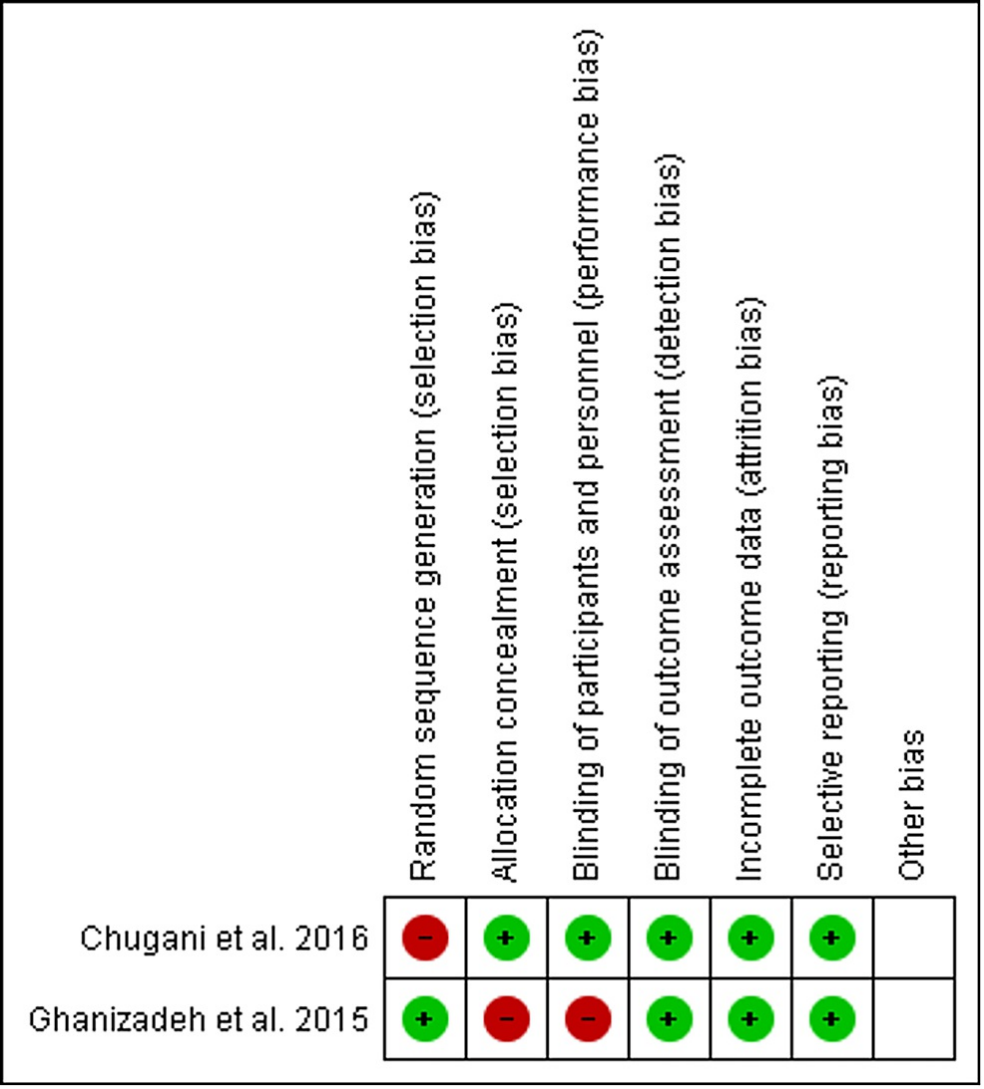
Risk of bias summary Presents all of the judgments made in evaluating the quality in a cross-tabulation of studies included in the review

**Figure 3:**
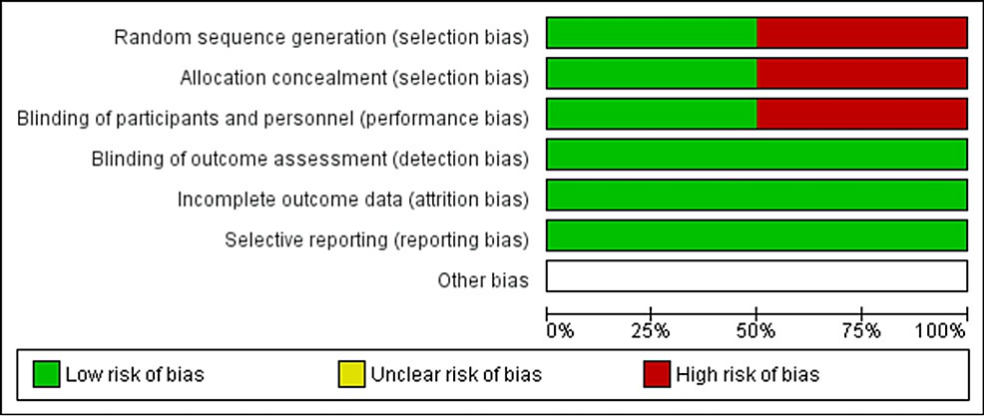
Risk of bias graph Illustrates the proportion of studies with each of the judgments ("low risk," "high risk," and "unclear risk" of bias) for each entry in the systematic review

## Review

RCTs

The first RCT [10] included 166 patients with ASD between the ages of two and six years. They either received oral buspirone 2.5 mg twice a day or oral buspirone 5 mg twice a day or a placebo for a duration of 24 weeks. The results indicated that although there was no difference in the ADOS composite score, the restricted and repetitive behaviors score showed significant improvement in the 2.5 mg buspirone group, compared to the placebo and 5 mg buspirone groups. PET scans were used to see if there were any foci of increased brain tryptophan metabolism, and additionally, serum levels of serotonin were checked to see if they correlated with response in children treated with buspirone. According to the study, oral buspirone at a relatively lower dose influenced core symptoms in younger children with ASD, though the overall score remained unchanged. The 2.5 mg group's superior performance was explained by the preferential stimulation of the presynaptic 5HT1A auto receptors at a lower dose compared to the postsynaptic receptors at a higher dose.

The second RCT conducted [11] had 40 children and adolescents between ages 4-17 years with irritability associated with ASD. The participants were randomly assigned to receive either risperidone plus placebo or risperidone plus buspirone (mean dose 6.7 mg/day) for eight weeks. Both groups showed significant improvements in irritability sub-scores. However, the buspirone group did better (scores declined from 25.7 to 16.3) compared to the placebo group (declined from 24.7 to 18.2). Buspirone and risperidone were both administered simultaneously, and although this study suggested that the combination of risperidone plus buspirone performed better than risperidone alone, some subjects may have simply responded better to risperidone alone even in the buspirone plus risperidone group. In addition, it had a relatively small sample size and was moderately biased. The author recommended that further studies be conducted with higher dosages of buspirone. A comparison with the buspirone-only group would have been helpful, but none was available.

Open-label studies

An open-label study [12] examined the safety and efficacy of 15-45 mg/day oral buspirone in managing anxiety and irritability in 26 children between the ages of 6- and 7 years with pervasive developmental disorder, based on DSM-III-R. Responders were followed for a period of up to 12 months after the open-label trial, with the treatment continuing for six to eight weeks. Twenty-one out of 22 participants completed the study, nine reported marked improvement, and seven reported moderate improvement in anxiety and irritability, as measured by the CGI scales.

Another open-label, cross-over trial [13] compared buspirone with fenfluramine or methylphenidate among four children with autistic disorder (DSM-III) aged 9 to 10.5 years. Three out of four individuals with mild to moderate intellectual disability and no history of seizures were included in two phases of four weeks each. The baseline readings were collected after two weeks of a drug-free washout period, and everyone was started on buspirone 5 mg BID for four weeks. This was followed by one week of washout after which the four individuals were switched to either MPH 2.5 mg to 5 mg BID or fenfluramine 10 mg/day to 20 mg per day for four weeks. Rating scales including the aberrant behavioral checklist, sensory-motor behavior checklist, and social awareness inventory were completed each week by parents and teachers who completed the Conners' teacher rating scale every week. Fifty percent showed a positive response with buspirone with hyperactivity, aggression, and stereotypy but, the response was indiscernible for rumination. Results were equivocal and inconclusive and recommended further clinical trials.

Another study was [14] conducted on a single child aged four years and 10 months over a period of three weeks to evaluate the safety and efficacy of buspirone hydrochloride in individuals with ASD and hyperactivity. The primary outcome measure was the number of performance tasks at school. The child received a placebo for three weeks and then a week of no drug treatment. Afterward, buspirone hydrochloride was commenced, which was found to be beneficial, but only with a modest 10% increase over the placebo.

Retrospective studies

The only retrospective study [15] reviewed 31 patients with high-functioning ASD and comorbid anxiety in the preceding year. They found significant improvement in anxiety symptoms in 58% of the patients and mild improvement in 29% of the patients who received buspirone at a mean dosage of 24.1 to 41.61 mg/day for an average of 125-272 days.

Discussion

This systematic review focuses on the safety and efficacy of buspirone in children and adolescents with ASD and its associated anxiety and irritability. There were only two RCTs, two open-label studies, one double-blind placebo control cross-over study, and one retrospective chart review. There was overall symptom improvement in all studies involving buspirone. Though these findings are consistent with anxiety disorders being a common comorbidity with ASD, the studies included in this review had different outcome measures, including core symptoms of autism, irritability, anxiety, and hyperactivity.

An evaluation of the efficacy and safety of oral buspirone in ASD lacks a high-powered double-blind RCT. Both RCTs concluded that low doses of buspirone were helpful and safe, but the data is insufficient. Meta-analysis could not be performed given the lack of homogeneity. The two RCTs were non-combinable, since one used buspirone alone, while the other used a buspirone plus risperidone combination that was started simultaneously. Therefore, additional RCTs comparing buspirone to placebo are needed.

Many studies have indicated that buspirone is a safe option, particularly in comparison to SSRIs, which have limited evidence in treating ASD and may worsen irritability due to the risk of behavioral activation. Considering this, buspirone could be a potentially safer alternative, especially for younger children, as it offers potential benefits with a lower likelihood of side effects.

Buspirone's mechanism of action is complex, including the activation of presynaptic 5-HT1A receptors at low doses and the activation of postsynaptic 5-HT1A receptors at higher doses. One RCT indicated that low doses (2.5 mg-5 mg) improved restricted and repetitive behaviors and interests. None of the medications have been found to be helpful in reducing core symptoms of ASD; thus, the improvement in these symptoms with buspirone is noteworthy.

Our review included irritability as a secondary outcome measure. Although one RCT found improvement in irritability, it did not have a buspirone-only group. It may be difficult to isolate the beneficial effects of buspirone from risperidone because the study included risperidone along with buspirone. There is potential for bias when examining the beneficial effects of buspirone on irritability, which is a feature in both internalizing and externalizing mental health disorders including ASD.

Although one of the RCTs used a low dose, both the RCTs used fixed-dose increments. It is unclear if the results would have changed if the variable dose titration schedules were studied.

An older open-label trial focused more on hyperactivity, thought to be one of the traits intrinsic to ASD. There was no evidence supporting buspirone's effectiveness in attention deficit hyperactivity disorder symptoms.

 Limitations

The studies do not separate age groups well, and there is no evidence of any difference in the response among high-functioning individuals with ASD as compared to the ones with lower intellectual functioning. The addition of buspirone in combination with other medications is not clearly understood. The duration of these trials was also shorter, and, therefore, a long duration of double-blinded placebo-controlled trials is needed.

## Conclusions

Limited data indicate that low-dose buspirone can be effective in treating restricted and repetitive behaviors of ASD given its action on the presynaptic 5HT1A receptor. Buspirone (dose ranging from 2.5 mg to 45 mg) may additionally be beneficial for anxiety, irritability, and hyperactivity associated with ASD. The tolerability profile of buspirone was favorable, with minimal side effects and no reports of behavioral activation.
